# Inequalities in prevalence of birth by caesarean section in Ghana from 1998-2014

**DOI:** 10.1186/s12884-022-04378-8

**Published:** 2022-01-22

**Authors:** Joshua Okyere, Henry Ofori Duah, Abdul-Aziz Seidu, Bright Opoku Ahinkorah, Eugene Budu

**Affiliations:** 1 Department of Population and Health, University of Cape Coast, Cape Coast, Ghana; 2 Research Department, FOCOS Orthopaedic Hospital, Accra, Ghana; 3 College of Public Health, Medical and Veterinary Sciences, James Cook University, Townsville, QLD Australia; 4 Department of Estate Management, Takoradi Technical University, Takoradi, Ghana; 5 School of Public Health, Faculty of Health, University of Technology Sydney, Ultimo, Australia

**Keywords:** Caesarean section, Inequality, Ghana, Demographic and health surveys, Global health

## Abstract

**Background:**

Caesarean section (CS) is an intervention to reduce maternal and perinatal mortality, for complicated pregnancy and labour. We analysed trends in the prevalence of birth by CS in Ghana from 1998 to 2014.

**Methods:**

Using the World Health Organization’s (WHO) Health Equity Assessment Toolkit (HEAT) software, data from the 1998-2014 Ghana Demographic and Health Surveys (GDHS) were analysed with respect of inequality in birth by CS. First, we disaggregated birth by CS by four equity stratifiers: wealth index, education, residence, and region. Second, we measured inequality through simple unweighted measures (Difference (D) and Ratio (R)) and complex weighted measures (Population Attributable Risk (PAR) and Population Attributable Fraction (PAF)). A 95% confidence interval was constructed for point estimates to measure statistical significance.

**Results:**

The proportion of women who underwent CS increased significantly between 1998 (4.0%) and 2014 (12.8%). Throughout the 16-year period, the proportion of women who gave birth by CS was positively skewed towards women in the highest wealth quintile (i.e poorest vs richest: 1.5% vs 13.0% in 1998 and 4.0% vs 27.9% in 2014), those with secondary education (no education vs secondary education: 1.8% vs 6.5% in 1998 and 5.7% vs 17.2% in 2014) and women in urban areas (rural vs urban 2.5% vs 8.5% in 1998 and 7.9% vs 18.8% in 2014). These disparities were evident in both complex weighted measures of inequality (PAF, PAR) and simple unweighted measures (D and R), although some uneven trends were observed. There were also regional disparities in birth by CS to the advantage of women in the Greater Accra Region over the years (PAR 7.72; 95% CI 5.86 to 9.58 in 1998 and PAR 10.07; 95% CI 8.87 to 11.27 in 2014).

**Conclusion:**

Ghana experienced disparities in the prevalence of births by CS, which increased over time between 1998 and 2014. Our findings indicate that more work needs to be done to ensure that all subpopulations that need medically necessary CS are given access to maternity care to reduce maternal and perinatal deaths. Nevertheless, given the potential complications with CS, we advocate that the intervention is only undertaken when medically indicated.

## Background

Maternal mortality is a public health concern across the globe for many years with the highest ratios occurring in resource-poor countries [1]. Particularly in low- and middle-income countries [LMICs], maternal mortality remains high despite significant advances that have been initiated to enhance maternal health outcomes [2]. Hence, in the year 2015, the world adopted 17 Sustainable Development Goals [SDGs] of which one, SDG-3, focused on improving maternal health. This goal envisions to reduce the global maternal mortality ratio (MMR) to less than 70 per 100,000 live births by 2030 [3]. In spite of this global commitment to mitigate MMR, it remains high in sub-Saharan Africa (sSA) [4]. WHO reports that about 66% of the world’s maternal deaths happens in sSA [5]. For that matter, it is imperative that there is equality and equity in the availability of basic and comprehensive emergency obstetric care such as caesarean section [CS] [3].

CS is regarded as life-saving intervention that ought to be accessible to all women who need it [6]. It is interesting to note the benefits of CS transcend beyond the mother’s health; it also enhances the health of the child [7]. Absolute and relative indications for CS vary and these include cephalopelvic disproportion, pelvic deformity, eclampsia, umbilical cord prolapse, threatening uterine rupture, placenta previa and fetal distress [8]. Inasmuch as there are benefits of birth by CS when demanded by indication, it is worth noting that birth by CS was also associated with severe adverse events including intraoperative and postoperative bleeding, as well as increased risks of maternal mortality, especially in regions like sSA where there are huge obstetric morbidities [8–10]. Nevertheless, there is a growing, unnecessary prevalence of elective CS as an alternative to spontaneous vaginal birth in recent years [11].

Generally, there is consensus in the literature that the optimal population-based CS rate should lie between 10 and 15% [11–13]. Very low population-based CS rates imply that women in need of CS in that country lack access [13, 14]. Nonetheless, there are inequities in the prevalence of CS in almost every country across the globe but more profound in LMICs, with some countries having as low as 3% and as high as 58% [6, 15, 16]. Evidence indicates that inequities in the prevalence of CS do not only occur between countries, but also within countries [16]. In a recent analysis of global, regional and national CS trends, Betran et al. found an increase in CS except for sSA [13]. However, further multi-country studies in Africa showed that indeed there were inequities within countries [16].

Ghana, like many sub-Saharan African countries, has a MMR of about 310 per 100,000 live births at the end of 2017 [17]. Given the fact that there are inequities in the prevalence of CS within countries, it is quintessential to focus attention on monitoring these in order to improve maternal health. To this end, we analysed trends in the prevalence of birth by CS in Ghana from 1998 to 2014.

## Methods

Data from Ghana Demographic and Health Surveys (GDHS) in 1998, 2003, 2008 and 2014 were analysed. GDHS forms part of global surveys implemented by Measure DHS in about 85 LMICs worldwide. Overarching focus of DHS is to collate information on children, women and men. Among the cardinal issues captured are CS, fertility and family planning. When sampling, selection of enumeration areas (EAs) is the first step and takes cognisance of rural and urban locations in Ghana. This is ensued by household selection in the EAs. The complete sampling procedure has been elaborated in the final reports of the 1998, 2003, 2008 and 2014 GDHS. The sample for this study consisted of women with live births in the 5 years preceding the survey who were answerable to questions pertaining to CS (*n* = 15,432). Focus of the analysis was on recent births of women of reproductive age.

### Variables of interest

Study outcome was whether mode of birth was by CS or not. Women who reported having given live birth by CS were categorised as “1”, whilst those without birth by CS were classified otherwise as “0”. Four stratifiers were used to assess inequality in births by CS: economic status measured by wealth quintile (quintile 1-5), education (no education, primary, secondary and above), residence (rural, urban) and region of residence (Western, Central, Greater Accra, Volta, Eastern, Ashanti, Brong Ahafo, Northern, Upper West, Upper East). Wealth index is derived by employing Principal Component Analysis (PCA). Education is measured by highest level of formal education completed.

### Statistical analysis

We used the 2019 updated WHO’s HEAT version 3.1 software for all analyses [18]. Estimates and confidence intervals of birth by caesarean section with respect to the aforementioned stratifiers were computed. Four measures were used to compute inequality namely Difference (D), Population Attributable risk (PAR), Population Attributable Fraction (PAF) and Ratio (R). Two of these are simple unweighted measures (D, R) and two are complex weighted measures (PAR, PAF). At the same time, R and PAF are relative measures whereas D and PAR are absolute measures. Summary measures were considered because WHO has indicated that both absolute and relative summary measures are essential for generating policy driven findings [18]. Unlike simple measures, the complex ones take size of categories inherent in a sub-population into account. WHO has extensively elaborated the procedure for generating summary measures [19].

## Results

### Trends in the prevalence of births by CS by different inequality dimensions, 1998-2014

**Fig. 1:**
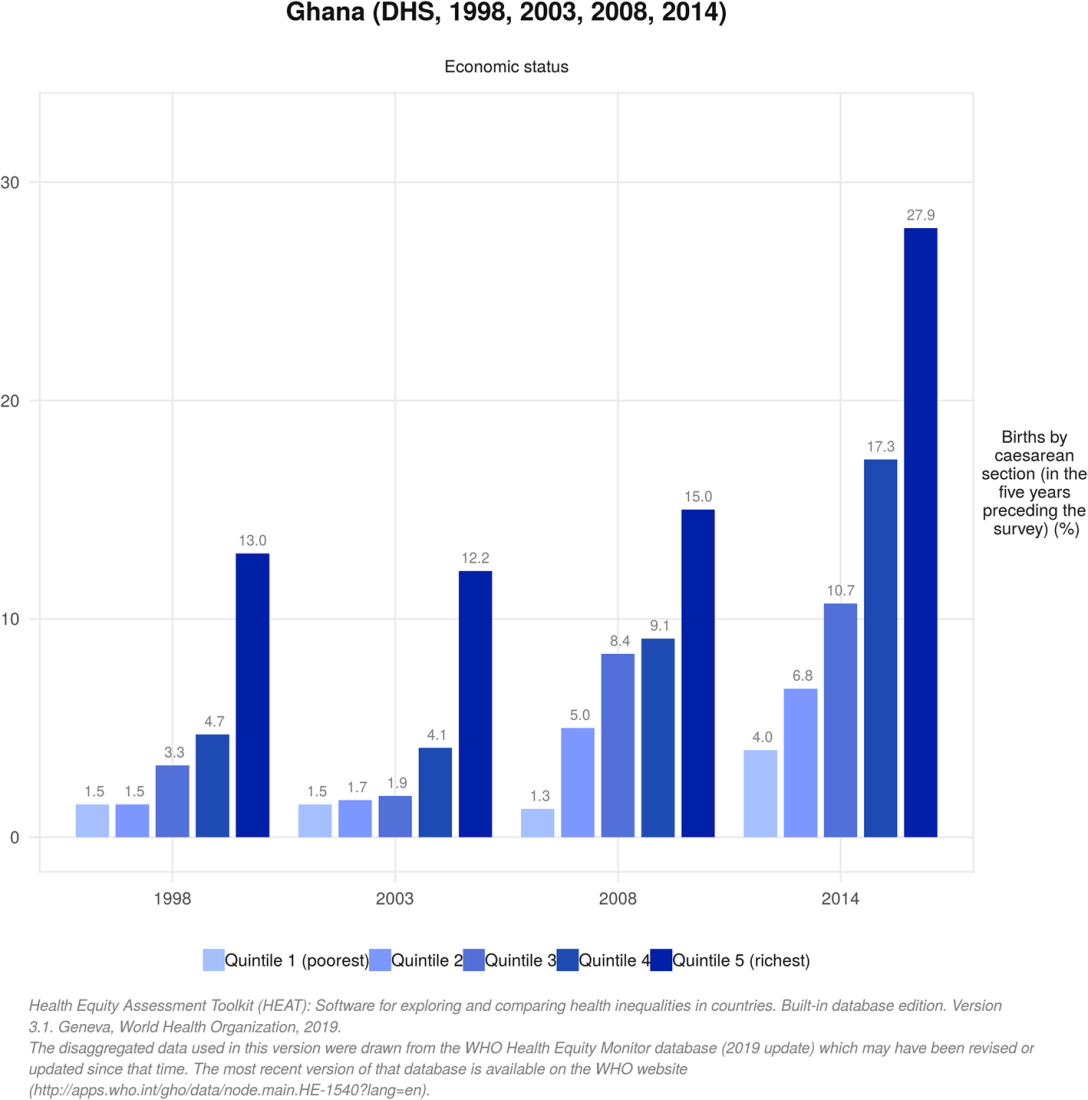
Trends in economic disparities in live births by caesarean section (CS) in Ghana from 1998 to 2014

**Fig. 2:**
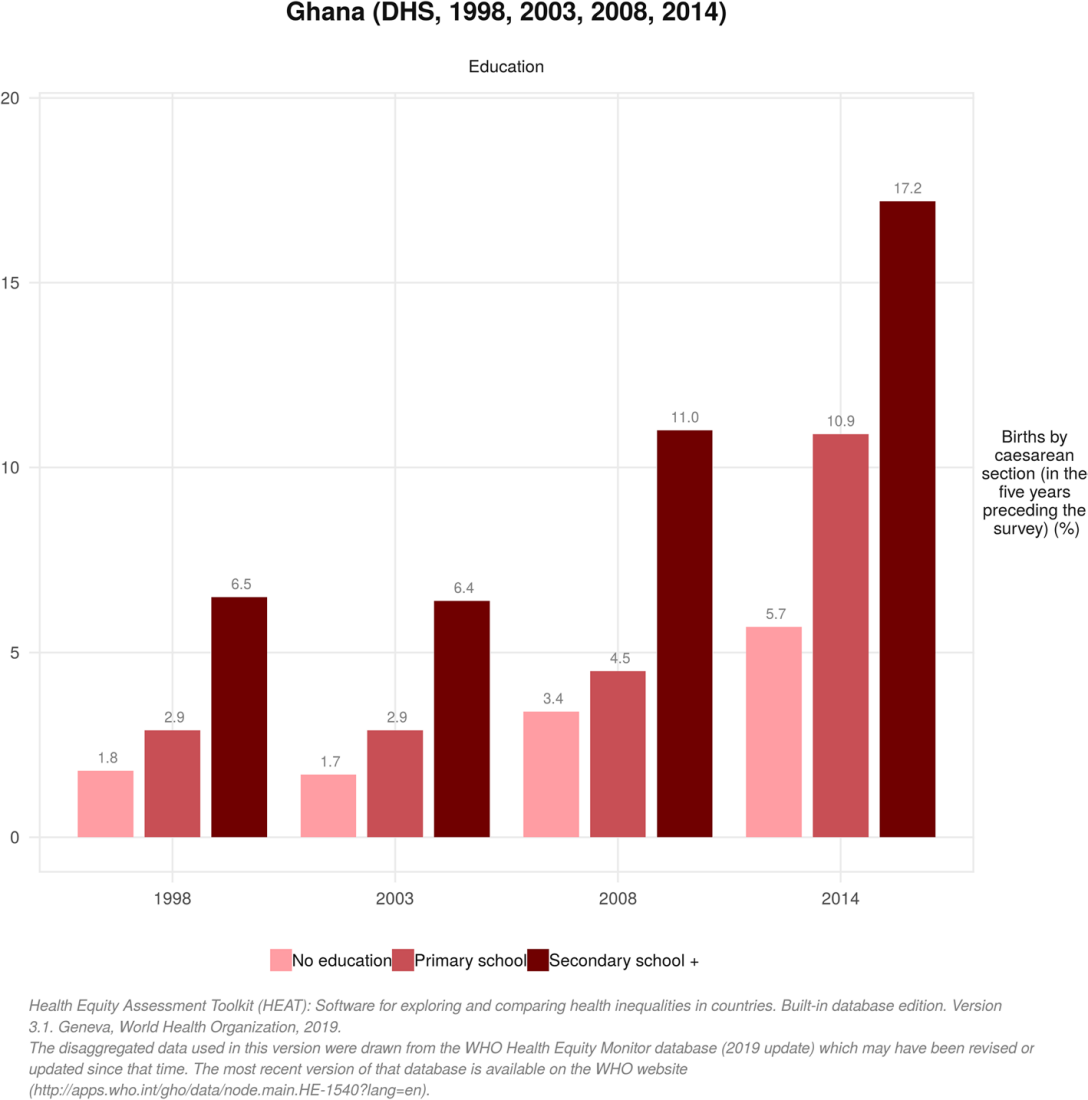
Trends in educational disparities in live births by caesarean section (CS) in Ghana from 1998 to 2014

**Fig. 3:**
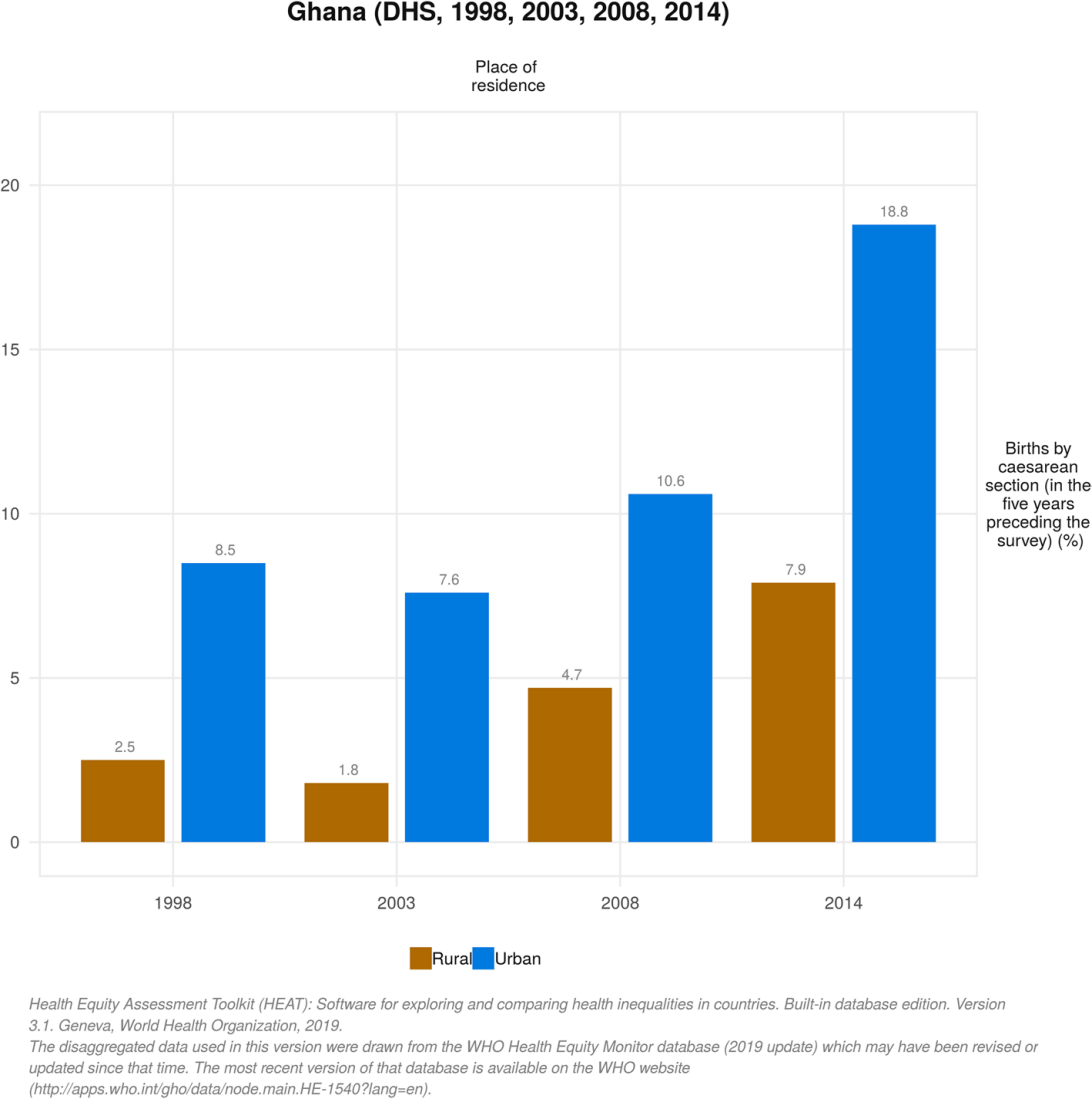
Trends in rural-urban disparities in live births by caesarean section (CS) in Ghana from 1998 to 2014

The proportion of women who underwent CS increased significantly between 1998 (4.0%) and 2014 (12.8%). Throughout the 16-year period, CS was skewed towards women in the highest wealth quintile and the gap increased as the years went by (i.e poorest vs richest: 1.5% vs 13.0% in 1998 and 4.0% vs 27.9% in 2014). Just as observed across economic status, births by CS were dominant among women who had secondary or higher education relative to those who had no formal education between 1998 [6.5, 95% CI = 5.13, 8.19 vs 1.8, 95% CI = 1.11, 2.92] and 2014 [17.2, 95% CI = 15.43, 19.18 vs 5.7, 95% CI = 4.26, 7.66]. The analysis also indicated that CS was highly concentrated among urban residents compared to their rural counterparts in 1998 [8.5% vs 2.5%] and increased to 18.8% vs 7.9% in 2014. Across the erstwhile ten adminstrative regions, we observed that CS was higher among women in the Greater Accra region across all different survey points. Meanwhile, as of 2014, the proportion of women in the Central Region who underwent CS had increased dramatically (15.7%) relative to the proportion 3.6% in 1998 (Table 1). Figures 1, 2 and 3 depict the economic, educational and rural-urban disparities in birth by CS in Ghana over the period 1998-2014 .

**Table 1:** Trends in the prevalence of births by caesarean section by different inequality dimensions, 1998-2014

Inequality dimension	1998 (3.97)		2003 (3.69)		2008 (6.90)		2014 (12.79)	
	Sample	Percentage [95% CI]	Sample	Percentage [95% CI]	Sample	Percentage [95% CI]	Sample	Percentage [95% CI]
**Economic status**								
Quintile 1 (poorest)	865	1.47 [0.80, 2.67]	941	1.48 [0.84, 2.60]	743	1.31 [0.72, 2.36]	1263	4.01 [2.99, 5.36]
Quintile 2	684	1.55 [0.76, 3.12]	809	1.73 [1.00, 2.97]	641	5.01 [3.11, 7.98]	1195	6.79 [4.97, 9.22]
Quintile 3	660	3.31 [1.98, 5.47]	720	1.90 [1.11, 3.24]	548	8.39 [5.56, 12.47]	1113	10.65 [8.59, 13.15]
Quintile 4	552	4.66 [3.11, 6.91]	616	4.14 [2.51, 6.75]	560	9.07 [6.41, 12.70]	1073	17.31 [13.80, 21.48]
Quintile 5 (richest)	431	12.96 [9.78, 16.98]	551	12.18 [8.84, 16.56]	414	14.97 [10.76, 20.44]	1048	27.88 [23.70, 32.48]
**Education**								
No education	1228	1.81 [1.11, 2.92]	1466	1.68 [1.06, 2.65]	951	3.43 [2.13, 5.47]	1561	5.72 [4.26, 7.66]
Primary school	648	2.93 [1.78, 4.78]	843	2.86 [1.68, 4.83]	722	4.52 [3.02, 6.72]	1140	10.85 [7.68, 15.14]
Secondary school +	1317	6.49 [5.13, 8.19]	1329	6.43 [4.84, 8.51]	1235	10.98 [8.83, 13.56]	2992	17.22 [15.43, 19.18]
**Place of residence**								
Rural	2420	2.52 [1.88, 3.36]	2435	1.77 [1.26, 2.49]	1805	4.67 [3.52, 6.17]	3131	7.89 [6.09, 10.17]
Urban	773	8.51 [6.62, 10.86]	1203	7.57 [5.67, 10.04]	1103	10.56 [8.19, 13.50]	2562	18.79 [16.59, 21.20]
**Region**								
Western Region	412	4.43 [2.24, 8.57]	366	2.24 [0.97, 5.06]	270	5.40 [2.82, 10.09]	573	14.57 [10.86, 19.28]
Central Region	379	3.58 [1.80, 7.00]	303	1.05 [0.32, 3.39]	292	10.01 [5.67, 17.06]	622	15.74 [11.00, 22.03]
Greater Accra Region	329	11.68 [9.06, 14.94]	389	11.98 [8.03, 17.50]	345	10.23 [6.59, 15.54]	880	22.86 [18.37, 28.08]
Volta Region	337	1.40 [0.44, 4.43]	298	3.67 [2.00, 6.63]	244	6.04 [3.41, 10.48]	435	8.82 [6.31, 12.19]
Eastern Region	430	5.68 [3.62, 8.80]	362	3.89 [1.87, 7.88]	254	7.62 [4.71, 12.09]	532	9.46 [6.63, 13.33]
Ashanti Region	513	2.27 [1.07, 4.73]	684	4.36 [2.50, 7.52]	544	10.65 [7.50, 14.90]	1064	15.61 [12.46, 19.38]
Brong Ahafo Region	259	3.14 [1.94, 5.05]	400	2.56 [1.23, 5.25]	271	4.91 [2.24, 10.45]	497	9.58 [7.48, 12.18]
Northern Region	232	1.41 [0.47, 4.14]	499	1.57 [0.65, 3.75]	455	2.55 [0.93, 6.76]	709	2.66 [1.53, 4.56]
Upper West Region	100	2.03 [0.66, 6.09]	117	1.85 [0.55, 6.04]	147	1.13 [0.22, 5.57]	226	7.56 [5.54, 10.25]
Upper East Region	198	1.05 [0.37, 2.89]	215	0.49 [0.06, 3.78]	81	3.46 [1.48, 7.91]	152	4.69 [3.07, 7.10]

*CI* Confidence Interval

### Inequality indices on factors associated with the prevalence of births by caesarean section, 1998-2014

In Table 2, absolute (D, PAR) and relative (R, PAF) summary measures are presented. A pattern of simple absolute (D) and relative (R) economic inequality in births by CS persisted throughout the period. The complex absolute measure (PAR) revealed increasing inequality from 1998 to 2014. The complex relative measure (PAF) showed an increase in disparity between 1998 and 2003 and subsequently a decline until 2014. Significant absolute and relative education-related inequality existed from 1998 to 2014 to the advantage of women with higher levels of education as revealed by all four summary measures. For instance, in 2014, R and PAR measures of 3.0% (95% CI; 2.07-3.95) and 4.4% (95% CI; 3.35-5.51), respectively, revealed significant inequality in births by CS to the disadvantage of women who had no formal education.

**Table 2:** Inequality indices estimates of the factors associated with the prevalence of births by caesarean section, 1998-2014 (%)

Inequality dimension	1998			2003			2008			2014		
	Estimate	LB	UB	Estimate	LB	UB	Estimate	LB	UB	Estimate	LB	UB
**Economic status**												
D	11.49	7.83	15.16	10.70	6.80	14.60	13.66	8.80	18.52	23.87	19.33	28.40
PAF	226.64	206.65	246.64	230.05	209.46	250.64	116.79	104.54	129.04	117.88	109.66	126.11
PAR	8.99	8.20	9.79	8.49	7.73	9.25	8.06	7.22	8.91	15.08	14.03	16.13
R	8.82	3.03	14.62	8.21	2.93	13.49	11.42	3.77	19.08	6.95	4.65	9.25
**Education**												
D	4.68	2.94	6.43	4.75	2.79	6.71	7.55	4.70	10.40	11.50	8.99	14.01
PAF	63.65	46.24	81.06	74.32	57.92	90.73	58.97	43.46	74.48	34.61	26.17	43.05
PAR	2.53	1.83	3.22	2.74	2.14	3.35	4.07	3.00	5.14	4.43	3.35	5.51
R	3.59	1.68	5.50	3.83	1.79	5.87	3.20	1.55	4.86	3.01	2.07	3.95
**Place of residence**												
D	5.99	3.77	8.20	5.80	3.57	8.04	5.88	2.94	8.82	10.90	7.84	13.96
PAF	114.39	103.08	125.71	105.21	93.46	116.97	52.87	42.18	63.56	46.84	40.91	52.76
PAR	4.54	4.09	4.99	3.88	3.45	4.32	3.65	2.91	4.39	5.99	5.23	6.75
R	3.38	2.10	4.66	4.28	2.38	6.18	2.26	1.41	3.10	2.38	1.71	3.06
**Region**												
D	10.64	7.54	13.73	11.49	6.72	16.25	9.52	5.44	13.60	20.21	15.16	25.26
PAF	194.50	147.59	241.41	224.53	181.34	267.71	54.25	19.31	89.19	78.70	69.29	88.11
PAR	7.72	5.86	9.58	8.29	6.69	9.88	3.75	1.33	6.16	10.07	8.87	11.27
R	11.17	0.54	22.87	24.28	26.36	74.92	9.43	6.16	25.01	8.61	3.59	13.63

*D* Difference, *R* Ratio, *PAR* Population Attributable Risk, *PAF* Population Attributable Fraction, *LB* Lower Bound, *UB* Upper Bound

The results also showed substantial rural-urban inequality in favour of urban residents throughout the 16-year period. In 1998, both complex absolute measure (PAR 4.5; 95% CI 4.09-4.99) and relative measure (PAF 114.4; 95% CI 103.08-125.71) showed significant urban-rural disparities in births by CS, however, the magnitude of the disparity declined in 2014 (PAF 46.8; 95% CI 40.91-52.76). In 1998, the complex absolute measure (D, PAR) revealed significant inequality in CS in favour of women in Greater Accra region as revealed by the PAR measure of 7.7% (95% CI 5.86-9.58). There was an uneven trend with this observation in PAR, having increased to 8.3% in 2003, then subsequently reducing to 3.8% in 2008 and rising again to 10.1% in 2014.

## Discussion

Socioeconomic inequalities in birth by CS still exist in Ghana and in fact have increased, despite the introduction of a free maternal health care policy in 2008 to bridge the gaps [20, 21]. The proportion of women who gave birth by CS increased substantially from 4.0% in 1998 to 12.8% in 2014 implying a percentage point increase of 8.8. Although a higher proportion of women may have benefitted from CS in 2014 compared to 1998, studies have shown that CS rates above 10% do not necessarily decrease maternal and perinatal mortality [5, 22, 23]. Recent evidence has shown that birth by CS increased risks of maternal mortality [10]. This is a reflection of Miller et al.’s “too little, too late” (TLTL) and “too much, too soon” (TMTS) care [24]. In their study, Miller et al. indicate that TMTS denotes the ‘unnecessary use of non-evidence-based interventions, as well as use of interventions that can be lifesaving when used appropriately, but harmful when applied routinely or overused’. Our findings of CS rates greater than 10% suggest the occurrence of TMTS and do not necessarily decrease maternal mortality. These show the existence of TLTL (more prevalent in lower wealth quintiles, rural areas and more peripheral regions) and TMTS (more prevalent in richer quintiles, urban areas and the Great Accra and Ashanti regions). Notwithstanding, our findings suggest that the observed increase in the prevalence of CS may be associated with some decreases in MMR from 501 per 100,000 live births in 1998 to 308 in 2017 [17]. Therefore, further studies will need to examine with empirical evidence whether MMR decrease is a result of increased CS prevalence in Ghana. The current prevalence of 12.8% is higher than the average CS rate of 7.3% in sSA and the average of 3% in West Africa [14, 25]. With this favourable trend per WHO standards, the challenge now lies on the equality gaps in access for all women in Ghana. It is also important to look at the increase in births by CS as a function of improved acceptability of CS over the years coupled with gradual improvement in health system readiness to perform CS. In addition, while this finding may be a good sign for women who need CS when it is medically indicated, we cannot rule out the possibility of rising cases of unnecessary CS which need further investigation.

We observed that the prevalence of CS was high among women who were in the highest wealth quintile compared to those in the lowest quintile and this gap increased as the years went by. This is supported by many other studies [7, 21, 26–31]. Caesarean sections are profitable for physicians, especially considering that it takes shorter time than vaginal birth, allowing many procedures to be performed within that time [32]. Unapproved fees charged by health professionals, indirect costs such as transport costs and other expenses outside the National Health Insurance Scheme (NHIS) and Free Maternal Healthcare Policy could serve as barriers for women in the lower wealth quintiles [33–35]. Women from high income families are more likely to afford additional costs associated with CS and thus are more open to elective CS as an alternative to spontaneous birth, which without a well-established indication is a form of “too much, too soon” [24]. A more worrying finding is the widening of the pro-rich inequalities as the years go by. This prompts for evaluation of programs and policies such as the NHIS established in 2003 and the Free Maternal Health Care Program inaugurated in 2008 by the government of Ghana with the aim of drastically reducing inequalities in health care including maternal health.

While previous studies in Egypt [36] and Ghana [37] have reported a low likelihood of CS among women with at least secondary school education, other studies in Ghana [21], China [26] and Bangladesh [29] reported findings in support of our study. A plausible explanation for this finding could be that women with higher education wrongly tend to perceive CS to be a safer way of childbirth, and partly because they believe it intereferes less with their work demands and leisure [38]. Another possible justification for the high patronage of CS by women with at least secondary school education could be related to increased autonomy and decision-making ability of educated women which allows them to recognise the relevance of CS when it is medically indicated [1, 4]. Women with low levels or no formal education are more likely to delay or refuse birth by CS, owing to fears of resulting pain and possible risks of infection, even when there are medical indications for CS, and would rather resort to spiritual interventions like prayers, hoping to eventually have vaginal birth [1, 39].

The fact that CS was more prevalent among women in urban areas from 1998 to 2014 was not surprising, because availability of advanced health facilities and skilled birth attendants are more present in urban areas. Women in rural areas often have less equipped and distant health facilities with few skilled birth attendants, making them more dependent on traditional births attendants. These lack technical expertise and license to perform CS, making access impossible [40]. Policies and programs must take into consideration this skewness and ensure that rural women are not discriminated when it comes to access to modern maternity care. The health system should thus have a well-functioning referral track in place.

Regional differences in CS showed the highest prevalence in the predominantly urban Greater Accra and Central Regions, while the Northern, Upper East, Upper West and Volta regions are predominantly rural and have lower CS-rates. It is also noteworthy that there are higher poverty rates in the three Northern, Upper East, and Upper West regions compared to the regions in the south [41]. The magnitude of inequalities between rural and urban areas with regard to CS prevalence, however, was reduced between 2003 and 2014, when the NHIS and Free Maternal Health Policy in Ghana were introduced. Nevertheless, one cannot conclude that this is mainly attributable to the introduction of the NHIS. Further studies could explore this association to inform the literature.

The findings of persistent inequities in CS among women in Ghana is a microcosm of the inequities in access to CS globally where extremes of access to CS have been documented with some women having it “too much, too soon” whiles others have it “too little”, “too late” [24]. While we advocate for the reduction of disparities in birth by CS, it is also worth noting that complications of CS exist. Women should undergo CS only when there is a genuine indication for it. Although we are aware of the growing popularity of elective CS among women, it is equally important to communicate those potential complications with pregnant women for informed decision making. Despite potential complications, we would recommend that women whose condition requires CS, get the necessary support for safe CS without any systemic disparities based on socio-economic status.

Given that we used existing data from the HEAT software, we were unable to account for singleton and multiple births separately. Moreover, while there was an overall increase in the proportion of birth by CS over the years, we saw an uneven trend in the dimension of inequalities signifying the need for sustained policy action despite transient challenges that may be encountered in advocacy for safe and appropriate birth by CS among women who have an indication for CS.

### Strengths and limitations

Our study has several strengths. First, to the best of our knowledge, this study is the first to examine inequalities in prevalence of CS in Ghana. Findings thus can be essential in guiding both policy and future research on CS in Ghana. Secondly, the use of both simple and complex measures of inequity contributes to the quality of our results as the limitations of one measure is compensated by combination with others. Thirdly, by presenting the findings for each subgroup, we provide a benchmark for the government to identify where attention is more needed in the midst of limited resources. Finally, using WHO’s HEAT software confirms the reliability of our findings. Nonetheless, there are some limitations that need to be acknowledged. Also, considering that the focus of our sample was on women with live births, our study excluded women with stillbirths who have been found to experience higher prevalence of CS. Given that the DHS estimates of the rate of birth by CS exclude women who underwent CS with a stillbirth in the samples, our findings may underreport the actual rate of birth by CS among women at reproductive age in Ghana.

We recommend the use of decomposition analysis to assess factors that could explain disparities in CS across various dimensions of inequality observed in this study. We were unable to differentiate emergency CS from elective CS which would have helped to situate our discussion in context. We were also unable to account for multiple pregnancies, which is an important risk factor for birth by CS. In addition, the study used secondary data without any influence over the selection and measurement of the variables. The effects of key variables such as place of birth (i.e., Health Centre, Polyclinic or Hospital) and type of health facility (public vs private) could not be investigated. Moreover, there has not been a recent DHS survey in Ghana making the last estimate in 2014 as the most recent one for discussion. Thus, the findings may not necessarily reflect current trends.

## Conclusions

Ghana experienced disparity in the proportion of birth by CS, which increased over time between 1998 and 2014. Our findings indicate that more work needs to be done to ensure that all subpopulations that need medically necessary CS are given access to maternity care to avoid unnecessary maternal and perinatal deaths. Nevertheless, provision of CS should also be done accurately to reduce deaths associated with complications of unnecessary CS. Given the increase in the proportion of birth by CS over the years, it is important that we begin to explore adverse events as well as effects on MMR in Ghana. Therefore, we recommend that future studies should concentrate on reasons of increased birth by CS and advocate the audit cycle in maternity care to reduce maternal and perinatal mortality and morbidity in Ghana.

## Data Availability

The datasets generated and/or analysed (including figures) are available in the WHO’s HEAT version 3 [https://www.who.int/gho/health_equity/assessment_toolkit/en/].

## Abbreviations

CS: Caesarean section
CI: Confidence Intervals
GDHS: Ghana Demographic and Health Survey
GHS: Ghana Health Service
GSS: Ghana Statistical Service
HEAT: Health Equity Assessment Toolkit
LMICs: Low and Middle Income Countries
MMR: Maternal mortality ratio
NHIS: National Health Insurance
PAF: Population Attributable Fraction
PAR: Population Attributable Risks
SDGs: Sustainable Development Goals
WHO: World Health Organization
